# *The Year-Book of Treatment* for 1888

**Published:** 1888-03

**Authors:** 


*The Year-Book of Treatment* for 1888. Pp. 336. London: CASSELL & CO.

*The Medical Annual.* 1888. Pp. 558. Bristol: WRIGHT.

These two admirable summaries of the past year's work do much to lighten the labours of the practitioner. They traverse the same ground for the most part, and may be compared side by side.

The Year-Book is, as heretofore, occasionally disfigured by the personal opinion of the reporter on the paper which he abstracts; this opinion is always uncalled for, and often impudent. There is nothing of this in the Annual. In the latter, abstracts are frequently supplemented, and most properly so, by abundant references to other papers or works dealing with the subject in hand. There is nothing of this in the Year-Book. The subjects in the Annual are arranged alphabetically, and have numerous cross references; in the Year-Book, the arrangement is into artificial groups, divided according to the casual studies of the abstractors, rather than the nature of the subjects. The most useful book for the practitioner is undoubtedly the Annual: the teacher who is somewhat lazy, and wants as much as possible of his reading and study done by proxy, may possibly prefer the Year-Book.

